# Impact of a School-Based Hygiene Promotion and Sanitation Intervention on Pupil Hand Contamination in Western Kenya: A Cluster Randomized Trial

**DOI:** 10.4269/ajtmh.2012.11-0633

**Published:** 2012-09-05

**Authors:** Leslie E. Greene, Matthew C. Freeman, Daniel Akoko, Shadi Saboori, Christine Moe, Richard Rheingans

**Affiliations:** Center for Global Safe Water, Department of Environmental Health, Rollins School of Public Health, Emory University, Atlanta, Georgia; Tropical Institute of Community Health and Development, Great Lakes University of Kisumu, Kenya; Center for Global Safe Water, Hubert Department of Global Health, Rollins School of Public Health, Emory University, Atlanta, Georgia; Department of Global and Environmental Health, University of Florida, Gainesville, Florida

## Abstract

Handwashing with soap effectively reduces exposure to diarrhea-causing pathogens. Interventions to improve hygiene and sanitation conditions in schools within low-income countries have gained increased attention; however, their impact on schoolchildren's exposure to fecal pathogens has not been established. Our trial examined whether a school-based water, sanitation, and hygiene intervention reduced *Escherichia coli* contamination on pupils' hands in western Kenya. A hygiene promotion and water treatment intervention did not reduce risk of *E. coli* presence (relative risk [RR] = 0.92, 95% confidence interval [CI] = 0.54–1.56); the addition of new latrines to intervention schools significantly increased risk among girls (RR = 2.63, 95% CI = 1.29–5.34), with a non-significant increase among boys (RR = 1.36, 95% CI = 0.74–2.49). Efforts to increase usage of school latrines by constructing new facilities may pose a risk to children in the absence of sufficient hygiene behavior change, daily provision of soap and water, and anal cleansing materials.

## Introduction

Globally, ∼1.3 million children under 5 years of age die each year due to diarrhea.^1^ This preventable illness is the leading cause of mortality in this age group in Africa.^2^ Handwashing with soap can reduce the risk of diarrhea by 42–48%^3^ and has been shown to effectively reduce pathogens of fecal origin on hands.^4^,^5^ Despite efforts to improve handwashing at key times to prevent fecal pathogen ingestion, studies from 13 low-income countries found that only 17% of child caregivers wash their hands with soap after defecation.^6^

There is increasing attention toward the impact of improved water, sanitation, and hygiene (WASH) conditions in low-income school settings, where poor conditions are thought to result in disease transmission among pupils and potentially to their younger siblings at home, who are most vulnerable to diarrhea-related mortality.^7^ Pupils may also act as agents of WASH behavior change in the community.^8^–^10^ Despite increased global efforts to improve school WASH infrastructure and behavior, lack of soap or handwashing at schools in low-income countries in particular has been cited as a major challenge, with some studies reporting as few as 2–7% providing soap for children.^11^–^15^

Some hygiene promotion trials in household or community settings have improved handwashing and reduced hand contamination among adults.^16^–^18^ However, others have not achieved these outcomes.^19^,^20^ Little is known whether school-based interventions can achieve substantial behavior change and pathogen reduction among pupils. Some school-based handwashing trials have had positive impacts on reported absence, without examining intermediate objective measures of improved hand hygiene or reduced pathogenic exposures.^8^,^21^–^24^ Although it is of great interest to know whether school WASH can reduce rates of diarrhea and absence among schoolchildren, intermediate objective measures of behavior and exposure provide important complementary data, and they can reveal whether reduced fecal exposure on hands is indeed the pathway by which higher level outcomes such as absenteeism may be reduced or whether other factors such as the appeal of having convenient, sanitary WASH facilities available in the school environment may be stronger determinants.

A number of school-based studies of hygiene interventions have shown improved knowledge and self-reported handwashing.^8^,^9^,^12^ However, neither are considered to be valid measures of behavior because of the strong tendency to report what is socially desirable and evidence that they correlate poorly with measured fecal indicator bacteria on hands.^6^,^16^,^25^,^26^ Alternative measurement of microbiological hand contamination can help to illustrate more objectively the degree to which a WASH intervention has reduced actual fecal exposures. We conducted a cluster-randomized control trial of two different school-based WASH interventions in western Kenya in which a sub-study assessed their impact on exposure to microbiological contamination on pupils' hands. The impact on school absence and diarrhea was the primary focus of the trial, and it was discovered that the intervention had no overall impact on school absence, but there was a significant reduction in absenteeism among girls in a geographical subset of schools. This sub-study provides additional insight into potential mechanisms by which the intervention may or may not have worked to achieve this higher order absence outcome.

## Methods

### Study design.

The purpose of this analysis was to assess the effect of a school-based WASH intervention on reducing fecal contamination on hands. This study was nested within a large cluster-randomized control trial of 135 public primary schools in four districts of Nyanza Province, Kenya. The goal of the large trial was to assess the impact of improved school WASH on health and educational outcomes of school children and their siblings.

Schools that exceeded the Government of Kenya (GoK) pupil/latrine ratio of 25:1 for girls and 30:1 for boys and that had a water source within 1 km during the dry season were eligible for the large trial.^27^ A complete description of the school selection procedures are described elsewhere.^24^ Schools were randomly selected and assigned to receive one of the following interventions with equivalent 1:1:1 allocation:

1. A hygiene promotion and water treatment (HP&WT) intervention that included buckets with lids and taps for handwashing and drinking water storage, and a year supply of WaterGuard, a locally available hypochlorite water disinfection solution. Teachers were trained on how to maintain drinking and handwashing facilities and to conduct behavior change promotion lessons with pupils through health clubs or other venues. The hygiene promotion curriculum addressed the importance of handwashing with soap at key times for diarrhea prevention and included training on proper handwashing techniques.
2. The same hygiene promotion and water treatment intervention with the added provision of up to seven new ventilated improved pit (VIP) latrines with concrete slabs to meet the GoK latrine ratio standards (Sanitation + HP&WT).
3. The control group, to receive the intervention at the conclusion of the study

Out of the 135 schools enrolled in the larger study, we randomly selected 17 intervention and 17 control schools where we collected hand rinse samples. Given the sub-study's original focus on the impact of the hygiene promotion activities, we pooled schools from both the HP&WT and Sanitation + HP&WT intervention arms for random selection, because both contained the hygiene promotion component, and we did not initially presume that the sanitation component would influence the results. As a sub-study of a secondary outcome, sample size was determined by the maximum processing capacity of our laboratory and logistical feasibility of data collection; no power and sample size calculations were performed. After baseline data collection in February and March 2007, the interventions were carried out by CARE Kenya and Water.org through a local partner Sustainable Aid in Africa International (SANA). After completion of the intervention, follow-up data collection occurred in September and October 2008.

### Pupil and school data collection.

At baseline and follow-up, trained enumerators arrived at schools unannounced, numbered pupils from grades 4 through 8 (typically 6 to 16 years of age) in enrollment rosters, and systematically sampled 25 from the rosters using a skip pattern proportional to the total number of pupils in these grades combined. Pupils completed an oral interview in the local Dholuo language about perceptions of school WASH conditions and personal WASH knowledge and practices. Questions included how often their school provided water and soap for handwashing, representing the opportunity to wash and develop hygiene habits. Enumerators asked pupils when they usually washed their hands and recorded whether they freely listed key occasions such as before eating and after defecating. Given the tendency for people to report desired behaviors, we considered this a measure of knowledge rather than practice. Latrine use habits and preferences were also assessed to determine potential changes in school latrine use over time.

The first 20 pupils who completed the interview and gave assent for sample collection contributed a hand rinse sample and were included in this study. Enumerators trained in laboratory methods guided each participant in placing his/her right hand in a 500 mL Whirl-Pak (Nasco, Fort Atkinson, WI.) bag containing 250 mL of phosphate buffered saline (PBS) solution. From outside the bag, enumerators assisted pupils in systematically agitating and rinsing all parts of the hand for 10 sec, ensuring full saturation. Bags were then sealed and placed in a cooler. Following sample collection, respondents were then led to a handwashing station and asked to demonstrate how they normally wash their hands. Enumerators recorded whether the participant successfully demonstrated key steps in handwashing, including the use of soap.

A school facility survey was also completed and included a teacher interview about typical WASH conditions at the school and structured observations of facilities such as presence of soap and water for handwashing and latrine cleanliness. All survey data were collected using personal digital assistants pre-programmed with questionnaires using Syware Visual CE v10 software (Cambridge, MA).

### Laboratory methods.

Hand rinse samples were transported at 4°C in coolers to a research laboratory at Great Lakes University of Kisumu and stored overnight at 4°C. The following morning, samples were analyzed for *Escherichia coli* by membrane filtration using standard methods and m-ColiBlue24 broth (Hach, Loveland, CO)^5^,^28^,^29^; for each sample, 1 and 10 mL volumes were filtered, and the plates were incubated at 44.5 ± 0.5°C for 24 hr.

Both dilutions were used to estimate concentrations of *E. coli* colony-forming units (CFU) per hand. When both plate counts were within the detection limit, concentrations were added and divided by the total volume filtered to determine the sample concentration. Plates exceeding 200 colonies were recorded as too numerous to count (TNTC). If one plate had zero colonies or TNTC, the other plate alone was used to estimate the concentration. Data were discarded from samples containing heavy background growth, atypical colonies, or samples processed at a time when negative control plates showed contamination. *Escherichia coli* concentrations were examined two ways: as presence of any detectable *E. coli* on hands versus absence, and as high contamination: ≥ 100 CFU/hand versus < 100 CFU/hand.

### Analysis.

For the primary impact analysis, we used individual pupil data and multivariable logistic regression using generalized estimating equations with a log-link function. Analysis was performed using the GENMOD procedure in SAS 9.2 (Cary, NC) to test the effect of the intervention on the risk of having *E. coli* present on hands. Models accounted for correlated observations within the school because of cluster sampling and pupil sampling weights. For the secondary analysis, we modeled the effect of the intervention on having high *E. coli* levels detected on hands. Although the degree of diarrhea risk associated with specific fecal contamination measures is unknown and varies by the type of pathogen and susceptibility of the individual, we assumed that *E. coli* loads of 100 CFU/hand or greater are likely indicative of more substantial exposure to enteric pathogens compared with having < 100 CFU/hand.

We tested the null hypothesis that there would be no difference in *E. coli* hand contamination among children in intervention schools compared with those in control schools. Our *a priori* assumption was that the results at both HP&WT and Sanitation + HP&WT intervention arms would not be different, because they both received the same hygiene promotion component of the program; therefore, our first analysis combined schools from both intervention arms. However, given the very different levels of contamination we measured on children's hands in each of the intervention arms, we decided to test the effect of each intervention separately. All preliminary models controlled for age, gender, and the interaction of the intervention with gender because of our finding that this program's impact on school absence was experienced differentially by gender and because of the high sectoral interest in the impact of school WASH on girls in particular.^7^,^24^ For simplicity of presentation we chose to show both gender-stratified and combined results for all models regardless of whether interaction was indicated. The impact of the intervention was modeled as the interaction between study arm (intervention versus control) and data collection round (baseline or follow-up), effectively measuring the difference between study arms while controlling for baseline values.^30^ In this way the change from baseline was compared between intervention and control.

To examine the context of the hand rinse results, we also compared the change in school WASH conditions and pupil behaviors (aggregated at the school level) from baseline to follow-up between the intervention and control schools using two-sample *t* tests. To create a single measure of overall school latrine cleanliness, we conducted a principal component analysis of observed ratings for odor, flies, and cleanliness. The resulting scale was then quintile ranked.

### Ethics.

School headmasters provided consent in *loco parentis*, and all pupil participants provided oral assent. Ethical approval was obtained from the Institutional Review Board of Emory University (Atlanta, GA). The Government of Kenya Ministries of Health, Water, and Education granted permission to conduct the trial.

## Results

Of the 17 intervention schools randomly selected for the study, 12 were in the HP&WT study arm, whereas five were in the Sanitation + HP&WT arm. At baseline and follow-up, we obtained 707 and 695 hand rinse samples, respectively, from 34 schools. Excluded were those for which negative control samples suggested possible laboratory-derived contamination of a batch (*N* = 20 baseline samples), those in which background or atypical growth rendered contamination counts unreliable (*N* = 46 at baseline; 11 at follow-up), and those that could not be matched to a pupil survey (*N* = 67 at baseline; 32 at follow-up). This resulted in a total of 574 baseline and 652 follow-up hand rinse samples from 34 schools.

### Changes in WASH conditions and behaviors.

At baseline, we observed that none of the schools had soap for handwashing on the day of data collection, and 93% (control) to 100% (Sanitation + HP&WT) of pupils claimed there was never soap at the school (Table 1). Hygiene conditions improved substantially in many intervention schools compared with control schools at follow-up. Though soap was not supplied by the intervention, we observed soap in 33% of HP&WT and 60% of Sanitation + HP&WT schools on the day of follow-up data collection compared with zero at control schools.

The average number of pupil-designated latrines in Sanitation + HP&WT schools increased from 5 to 14, indicating some constructed additional latrines above those provided by the intervention (data not shown in table). The percentage that was VIP latrines increased from 57% to 82%. The average number of pupil-designated latrines in HP&WT schools was 7 at baseline and follow-up, with those meeting VIP latrine standards decreasing from 51% to 9%. Pupil latrines in control schools slightly increased from 6 to 7. Those with VIP features decreased from 46% to 33% at follow-up (data not shown). The number of pupils per latrine at baseline exceeded the GoK standard in all study arms. This ratio dropped substantially in Sanitation + HP&WT schools for both genders. Both observed and pupil-reported measures of latrine cleanliness suggest significant improvement in conditions of latrines in Sanitation + HP&WT schools. Pupil-reported comfort and use of school latrines also improved in this study arm.

### Hand contamination.

In both intervention arms combined, 41% of pupils (95% confidence interval [CI] = 32–50%) had any *E. coli* present on their hands at baseline. This increased to 68% (95% CI = 52–83%) at follow-up (data not shown). The frequency of children with high levels of contamination in combined intervention schools increased from 26% (95% CI = 17–35%) at baseline to 57% (95% CI = 39–75%) at follow-up.

#### Hand contamination by intervention arm.

**Figure 1.**
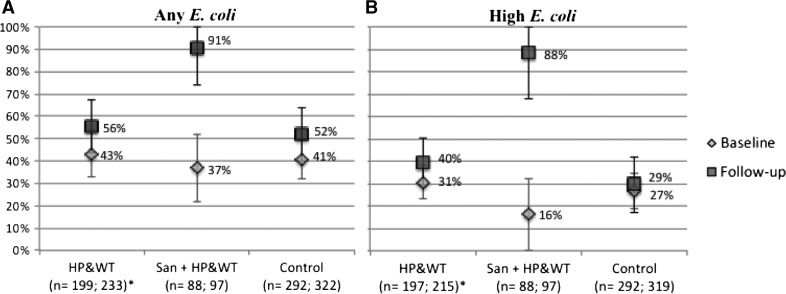
(**A** and **B**) Percentage of pupils with presence of any and high levels (≥ 100 colony-forming units (CFU)/hand) of *Escherichia coli* on their hands at schools receiving hygiene promotion and water treatment (HP&WT), additional sanitation (San + HP&WT), and control schools at baseline and follow-up. *n for baseline; follow-up

Figure 1A and B shows changes in both the presence of any and the presence of high hand contamination according to each intervention arm. Schools in the HP&WT and control groups experienced similar, slight increases in the percentage of pupils with any *E. coli* present on their hands, whereas the Sanitation + HP&WT schools experienced a greater increase from 37% at baseline to 91% at follow-up. The proportion of children with high levels of *E. coli* contamination stayed constant in the control schools, increased slightly in HP&WT schools, and increased from 16% to 88% in Sanitation + HP&WT schools.

### Association of hand contamination with intervention.

We examined the impact of the intervention on pupil hand contamination. Per our initial study protocol, we first compared the change in presence of any *E. coli* hand contamination between pupils attending schools in either intervention arm to those in the control schools. The intervention had no impact on the presence of any *E. coli* hand contamination (relative risk [RR] = 1.1, 95% CI = 0.7–1.8, *P* = 0.72) (data not shown). There was also no impact on having high levels of contamination (RR = 1.5 95% CI = 0.7–3.2, *P* = 0.34).

There was marginally significant evidence of gender as an effect modifier for the any *E. coli* outcome (*P* = 0.07). Among girls there was a significant 79% increase in risk of having *E. coli* hand contamination (RR = 1.8, 95% CI = 1.1–3.0, *P* = 0.03), whereas there was no significant change for boys (RR = 1.0, 95% CI = 0.5–1.7, *P* = 0.89). For the high contamination outcome, there was strong evidence of gender as an effect modifier (*P* < 0.001). Girls who attended an intervention school experienced a 4.2 times increased risk of having high *E. coli* hand contamination at follow-up (RR = 4.2, 95% CI = 1.9–9.6, *P* < 0.001), whereas there was no significant impact on boys (RR = 1.1, 95% CI = 0.5–2.6, *P* = 0.79).

#### Relative risk of contamination by intervention arm.

Given the substantially different hand contamination levels observed at schools in different intervention arms, we conducted a secondary regression analysis to examine the separate intervention effects for each arm (Table 2). Evidence of significant interaction by gender was found in our model of any *E. coli* with the Sanitation + HP&WT intervention as well as our models for high *E. coli* with both intervention arms. In schools that received the HP&WT intervention only, there was no impact on risk of having any *E. coli* hand contamination; however, among girls, the risk of having high levels of *E. coli* was 2.2 times higher than it was for girls in control schools (95% CI = 1.2–3.9). In contrast, among boys there was a 26% reduction in risk of having high *E. coli* contamination levels on hands, but this result was not significant (RR = 0.7, 95% CI = 0.3–1.7).

Children who attended schools that received a Sanitation + HP&WT intervention experienced an increased risk of any *E. coli* and high *E. coli* contamination on their hands. The risk of having any *E. coli* was 2.6 and 1.4 times higher among girls and boys, respectively, compared with those who attended control schools, although the change for boys was not statistically significant. The risk of having high *E. coli* contamination levels on hands was 9.8 times higher for girls (95% CI = 2.4–39.6) and 2.6 times higher for boys (95% CI = 0.8–8.5) in the Sanitation + HP&WT intervention schools compared with children in the control schools.

### Additional assessment by gender.

**Figure 2.**
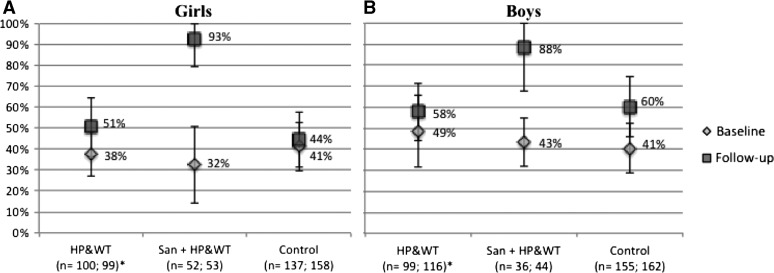
(**A** and **B**) Percentage of pupils with presence of any *Escherichia coli* on their hands, by gender, at schools receiving hygiene promotion and water treatment (HP&WT), additional sanitation (San + HP&WT), and control schools at baseline and follow-up. *n for baseline; follow-up.

**Figure 3.**
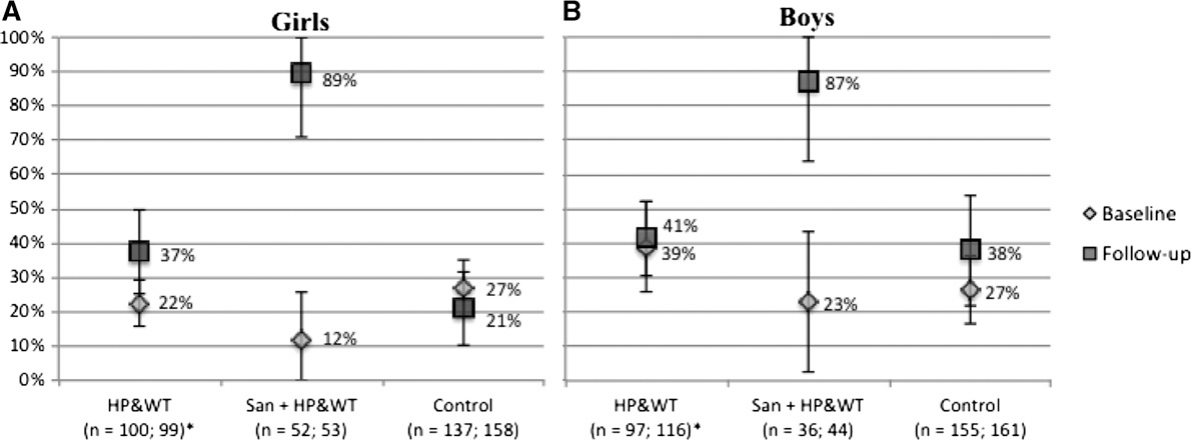
(**A** and **B**) Percentage of pupils with high levels (≥ 100 colony-forming units (CFU)/hand) of *Escherichia coli* on their hands, by gender, at schools receiving hygiene promotion and water treatment (HP&WT), additional sanitation (San + HP&WT), and control schools at baseline and follow-up. *n for baseline; follow-up.

We further examined levels of hand contamination by gender. Although the proportion of girls with any hand contamination present stayed relatively constant at control schools, there was a 13% point increase in HP&WT schools and a 60% point increase in Sanitation + HP&WT schools (Figure 2A and B). Among boys, there was a 20% point increase in control schools compared with a 9- and 45-point rise for boys in the HP&WT and Sanitation + HP&WT schools, respectively. The contrast between boys and girls was greater for the high contamination outcome (Figure 3A and B).

#### Behavioral and attitudinal changes by gender.

To understand potential explanations for the results, we also examined patterns of changes in behavioral and attitudinal WASH indicators by gender (Table 3). In Sanitation + HP&WT schools, there was a significant 17% point increase in girls claiming they always defecate at school when necessary compared with a 12% point decrease at control schools. There was a less pronounced, marginally significant 6% point increase in reported latrine use among girls at HP&WT schools. There were no significant changes in reported or demonstrated handwashing practices for either gender in either intervention arm.

## Discussion

Our study found that an intervention consisting of school-based hygiene promotion and water treatment did not impact pupils' risk of having *E. coli* hand contamination. However, girls had a significantly increased risk of having high levels of *E. coli* contamination on their hands. The addition of new sanitation facilities to the HP&WT intervention greatly increased children's risk of having any *E. coli* and high levels of *E. coli* on their hands. This effect was significant and of the highest magnitude among girls. These findings suggest a lack of sufficient improvement in handwashing behavior in intervention schools coupled with an undetermined source of increased contamination risk in Sanitation + HP&WT schools.

There are several potential reasons for these unexpected results. Hand contamination levels are likely to vary depending on several factors, including the degree to which hands were contaminated during defecation, whether the individual washed his/her hands with water and soap before sample collection, the quality and duration of handwashing, the level of environmental contamination with feces on surfaces, and the length of time since last handwashing or defecation. Given the rapid decline in *E. coli* survival on skin, we assume that the contamination we detected on hands was recently acquired while at school.^16^,^31^ Our data show that handwashing materials were more frequently available following the intervention. Therefore, children in the intervention schools had increased opportunity to wash their hands, although some schools did not have handwashing materials on the day of data collection. Alternative behavioral indicators suggest that pupils may not have increased their practice of regular handwashing or of thorough handwashing. There was no significant change in the percentage of girls or boys that used soap during a handwashing demonstration, which, in one study, was shown to be the closest correlate (albeit imperfect and prone to overestimation) with observed behavior among caregivers in Indian households compared with other self-reported indicators.^25^ The hygiene promotion intervention relied on a simple curriculum and training of teachers to pass on messages to pupils, which may not have been sufficient to change behavior. There is evidence that health message-based hygiene promotion efforts alone are not always sufficient to motivate behavior change among adults in developing countries, but it is not known whether this strategy improves hygiene practices among children^6^,^19^; an evaluation of an intervention in Kenyan schools found no evidence that teacher trainings and school health club activities improved handwashing behavior.^13^

The sharp increase of contamination in Sanitation + HP&WT schools may be caused by increased usage of school latrines for defecation without concurrent improvement in hand hygiene after using them. Usage of school toilets is associated with their level of cleanliness^12^,^13^; we observed, and pupils confirmed, a perception that latrine cleanliness in the Sanitation + HP&WT schools improved significantly. Additionally, indicators of comfort in using school latrines in this intervention group suggest that pupils probably increased their usage of the new latrines, which may have provided an appealing alternative to their home latrines or lack thereof. Kenyans tend to habitually defecate in the morning upon waking up,^32^ but children in Sanitation + HP&WT schools may have chosen to delay defecation until they arrived at school. We did not conduct observations that would detect such changes in defecation habits. Girls in these schools reported a greater increase in usage of the latrines than boys, which may in part explain the greater proportion of girls with *E. coli* hand contamination in these intervention schools if this is indeed a risk factor. However, there are likely other unmeasured behavioral factors among girls and boys that may explain the increased risk of contamination in both intervention arms. This merits future research. We did not collect data to determine whether girls were engaged in latrine cleaning more often than boys; however, anecdotal evidence from numerous school visits indicates that both girls and boys in Nyanza Province schools tend to be equally responsible for cleaning the latrines designated to their gender. It is important to note that separate analysis of results for each intervention arm was an unplanned exploratory analysis and should therefore be interpreted cautiously.

Anal cleansing materials, such as toilet paper, are almost never provided by Kenyan primary schools and were not provided as part of the intervention. Lack of toilet paper at schools and dirty toilets have been shown to be associated with diarrhea.^33^ A study by McMahon and colleagues^34^ was performed following this trial, which examined anal cleansing habits among pupils in our study area. It was discovered that children in this area use a variety of materials for anal cleansing after defecation including leaves, paper from schoolbooks, stones, corncobs, and their hands. Toilet tissue is seldom used because of the high cost, or for some a lack of awareness, and cleansing with water is not commonly practiced among most people in the local Luo culture. Some school children explained that commonly used materials are often inadequate and feared disease transmission as a result. Anal cleansing habits did not appear to differ by gender. According to anecdotal reports, in some cases, smaller children without anal cleansing materials may smear fecal matter on the walls of the latrine. Our study did not include observations of these conditions, but this may be another location where hand contamination can occur. If the lack of sufficient anal cleansing materials at school leaves a child with extremely high levels of fecal contamination on his/her hands, a cursory handwashing—particularly if done without the use of soap–may not remove all pathogens. We did not observe the proximity of handwashing facilities to latrines or the time of day when soap and water for washing were set out. It is possible that they were not available before the start of classes, when pupils may have been using the latrines. Given the lack of anal cleansing materials, if pupils in Sanitation + HP&WT schools used school latrines more often, and if their handwashing practices did not improve substantially, it is conceivable that they would have an increased risk of fecal contamination on their hands. Further research should be conducted to test these hypotheses.

Environmental contamination on surfaces may have also contributed to soiled hands in our study. This appeared to be a factor in research by Ram and colleagues^35^ in Bangladesh, where 80% of women's hands were found to be contaminated 2 hours after thorough handwashing with soap. A study among street vendors in Guatemala observed a similar trend.^20^ Likewise, Pickering and colleagues^36^ discovered that Tanzanian women had substantial hand recontamination following observed typical household activities, suggesting that environmental contamination with feces was pervasive. One trial found that cleaning desks and other surfaces in a United States elementary school reduced episodes of gastrointestinal illness.^37^ Environmental contamination should be explored in future studies in low-income settings.

Measurement of diarrhea or absence more directly explain the impact of the intervention on key outcomes of interest, and these data are reported elsewhere.^24^ This study sheds light on the potential mechanism by which this school WASH intervention might have influenced rates of illness and absence. Results from the main impact study revealed that the impact of Sanitation + HP&WT on pupil absence was no different relative to controls than in schools that received HP&WT without sanitation.^24^ Though it is difficult to draw a direct link between these hand rinse results and our measure of self-reported absence in the prior 2 weeks, this suggests a possibility that some of the benefit conferred by new latrines at school may have been offset by increased illness from elevated risk of hand contamination. The main study findings also suggested a reduction in absenteeism among girls.^24^ Although comparison of the two indicators is challenging, the increased hand contamination among girls in contrast to this finding suggests that perhaps only a fraction of diarrhea and consequent absenteeism may be attributable to hand contamination and that patterns of school absence may relate in part to the value placed on latrines and handwashing facilities as amenities. Our data show that girls in particular may have been drawn to the amenity value of school latrines, given indictors of acceptability and usage in Sanitation + HP&WT schools (Table 3).

### Limitations.

Although other studies have measured the impact of school WASH interventions on hygiene awareness or reported behavior, to our knowledge, this is the first study that examines the impact on pupil hand contamination in low-income settings. However, the limitations of our study should be considered. Although we can informally assess the influence of specific WASH conditions and behaviors on hand contamination outcomes, these factors could not be tested in regression models, as our analysis was conducted according to intention to treat (i.e., intervention status), and such conditions and behaviors are presumed to be artifacts of the intervention itself. In addition, we did not have a direct measure of handwashing behavior to confirm whether lack of change in handwashing may be an explanation for the findings.

Hand rinse sampling has been shown to be a valid measure of handwashing effectiveness.^5^,^16^,^18^ However, some studies suggest it is not likely to accurately reflect whether subjects washed their hands, particularly because of the high variability found in repeated hand rinse measures and lack of correlation with other hand hygiene indicators, potentially a result of environmental recontamination.^29^,^35^ It is also important to note that our data do not quantify risk of diarrhea, as there is no well-defined relationship between levels of fecal indicator bacteria on hands and risk of enteric illness, and there is considerable variability in individual host susceptibility and pathogen virulence. Ideally, children would not be exposed to any fecal pathogens on their hands, and our presence/absence indicator was chosen to reflect this. Although *E. coli* have been used in similar studies, it has been suggested that fecal streptococci, *Clostridium perfringens*, or enterococci are better fecal indicator bacteria because of their longer survival on skin.^17^,^36^,^38^,^39^ We chose to incubate laboratory samples at 44.5°C rather than the 35°C temperature recommended for m-ColiBlue24 medium to reduce the growth of non-specific background colonies to facilitate more accurate counting of the target *E. coli* colonies. The higher incubation temperature has been previously validated for this medium in tropical water samples, which we processed simultaneously with hand rinse samples for a different component of the study.^40^ Our data may be a conservative estimate of *E. coli* concentrations because of the higher incubation temperatures and cannot be directly compared with other studies that enumerate *E. coli* at 35°. Finally, the small number of schools sampled from the Sanitation + HP&WT intervention arm (*N* = 5) limited the precision of the results.

## Conclusions

We found no reduction in hand contamination in intervention schools as originally hypothesized. We did, however, find a dramatic increase in hand contamination among children in the Sanitation + HP &WT schools. Though the mechanism for these findings is not certain, our results suggest that efforts to increase the quantity of school latrines may pose a risk to children in absence of actual hygiene behavior change, daily provision of soap and water prior to children's arrival at school, and provision of anal cleansing materials to prevent hand contamination. In Kenya and other countries, the sustainability of soap in schools is a challenge and has been attributed to insufficient funds, lack of motivation from teachers, or unclear roles and responsibilities.^11^,^13^,^15^,^41^ Approaches that overcome these barriers are needed as a first step to improve school hygiene. The effectiveness of various behavior change education strategies for schoolchildren should also be evaluated in future studies. Research that combines objective hand rinse data with observation methods is needed to more closely examine the relationship between actual latrine usage, handwashing behavior, and hand contamination. Potential gender differences in sanitation and hygiene behaviors need to be better understood so that WASH intervention strategies can be appropriately developed to address the unique needs of both girls and boys. Future WASH intervention trials should attempt to better understand the specific mechanisms by which school attendance is impacted, as assumptions about disease reduction may not hold. In addition, our findings point to a need for increased attention to the role that anal cleansing materials may play in the prevention of hand contamination. This topic has been largely ignored in school WASH programs. Finally, studies that assess the impact of surface cleaning in schools in low-income settings are needed. In light of the growing global push for improving sanitation coverage in schools, our findings should be carefully considered and explored further.

## Figures and Tables

**Table 1:** School and pupil characteristics at intervention versus control schools at baseline and follow-up

	HP&WT*			Sanitation + HP&WT*			Control	
	Baseline	Follow-up		Baseline	Follow-up		Baseline	Follow-up
	(*N* = 12)			(*N* = 5)			(*N* = 17)	
	Mean/% (SE)‡	Mean/% (SE)‡	*P*†	Mean/% (SE)‡	Mean/% (SE)‡	*P*‡	Mean/% (SE)‡	Mean/% (SE)‡
School characteristics								
Mean school size	350 (38)	362 (42)	0.54	383 (73)	421 (77)	0.42	261 (14)	282 (17)
% with observed water for handwashing (n)	8 (1)	83 (10)	< **0.01**	0 (0)	80 (4)	< **0.01**	6 (1)	6 (1)
% with observed soap (n)	0 (0)	33 (4)	**0.01**	0 (0)	60 (3)	<**0.001**	0 (0)	0 (0)
Mean girls per latrine	55 (5)	53 (6)	0.81	105 (32)	31 (4)	0.10	52 (8)	45 (4)
Mean boys per latrine	65 (9)	57 (7)	0.49	130 (30)	28 (4)	**0.04**	62 (8)	52 (6)
Mean % latrine banks with feces observed on slab	6 (4)	6 (4)	0.36	27 (15)	0 (0)	**0.05**	12 (6)	20 (7)
Mean cleanliness quintile ranking of latrines§	4 (0)	3 (0)	0.58	3 (0)	4 (0)	< **0.01**	4 (0)	2 (0)
Pupil characteristics¶								
% females	51 (4)	45 (3)	0.15	59 (4)	55 (4)	0.30	47 (3)	50 (4)
mean age	13 (0)	13 (0)	0.89	14 (0)	13 (0)	0.43	13 (0)	13 (0)
% reported water always available at school for handwashing	16 (7)	54 (8)	0.07	23 (11)	86 (10)	0.10	11 (4)	21 (7)
% reported soap always available at school	0 (0)	34 (8)	**0.01**	0 (0)	47 (6)	**0.02**	4 (4)	5 (3)
% reported soap never available at school	99 (1)	27 (6)	<**0.001**	100 (0)	9 (2)	**0.02**	93 (4)	92 (5)
% reported washing hands after using a latrine	78 (5)	87 (2)	0.11	83 (5)	89 (5)	0.18	82 (3)	81 (3)
% used soap in handwashing demonstration	71 (5)	78 (7)	0.75	85 (3)	81 (8)	0.62	82 (5)	84 (3)
% reported discomfort using school latrines	47 (7)	28 (5)	0.24	58 (11)	15 (5)	**0.04**	51 (7)	48 (4)
% reported always defecating at school as needed	76 (6)	82 (4)	0.13	80 (6)	90 (2)	0.10	79 (4)	70 (4)
% reported never defecating at school	3 (1)	2 (1)	**0.04**	5 (2)	1 (1)	0.08	4 (1)	9 (2)
% reported school latrines usually very dirty	23 (6)	11 (4)	0.38	52 (10)	4 (3)	**0.01**	28 (6)	25 (7)

* HP&WT schools received intervention with hygiene promotion and water treatment. Sanitation + HP&WT schools received the same, plus additional latrines.

† *P* value of *t* test (or χ^2^ for schools' observed soap, water) comparing difference from baseline to follow-up between intervention and control groups.

‡ Unless otherwise noted.

§ Observed levels of odor, flies, and cleanliness were submitted to a principal components analysis and quintile-ranked. Higher numbers represent better conditions.

¶ Pupil results are school-aggregated values, adjusted for cluster sampling and unequal probability of pupil selection. At HP&WT, Sanitation + HP&WT, and control schools, respectively, figures are composed of *N* = 204; 89; 296 pupils at baseline, and *N* = 219; 97; 325 pupils at follow-up.

**Table 2:** Relative risk of having any or high *Escherichia coli* hand contamination for children attending schools that received hygiene promotion and water treatment or an intervention with additional sanitation versus controls*

	HP&WT†				Sanitation + HP&WT†			
	n	Relative risk	(95% CI)	*P*‡	n	Relative risk	(95% CI)	*P*‡
Any *E. coli*								
Combined	1026	0.92	(0.54, 1.56)	0.75	797	1.61	(0.86, 3.01)	0.14
Girls	494	1.27	(0.75, 2.14)	0.38	400	2.63	(1.29, 5.34)	**< 0.01**
Boys	532	0.79	(0.42, 1.51)	0.48	397	1.36	(0.74, 2.49)	0.33
High *E. coli*§								
Combined	1023	0.97	(0.48, 1.94)	0.92	796	3.69	(1.08, 12.60)	**0.04**
Girls	494	2.18	(1.21, 3.94)	**< 0.01**	400	9.75	(2.40, 39.56)	**< 0.01**
Boys	529	0.74	(0.33, 1.65)	0.46	396	2.60	(0.80, 8.48)	0.11

* Combined results control for age, gender, and interaction of gender with the intervention. All stratified models control for age.

† HP&WT schools received intervention with hygiene promotion and water treatment. Sanitation + HP&WT schools received the same, plus additional latrines.

‡ χ^2^ probability.

§ ≥ 100 colony-forming units (CFU)/hand.

**Table 3:** Changes from baseline to follow-up in water, sanitation, and hygiene (WASH) attitudes and behaviors among girls and boys attending intervention versus control schools

	HP&WT*				Sanitation + HP&WT*				Control	
	Girls		Boys		Girls		Boys		Girls	Boys
Change in pupil characteristics†	%	*P*‡	%	*P*‡	%	*P*‡	%	*P*‡	%	%
Report discomfort using school latrines	−19	0.98	−18	0.11	−45	**0.02**	−39	**0.03**	−10	5.8
Report always defecating at school when needed	6	0.08	5	0.18	17	< **0.01**	1	0.39	−12	−9.7
Report never defecating at school	−2	**0.01**	1	0.43	−7	< **0.01**	0	0.25	7	4.1
Report washing hands after using a latrine	5	0.29	7	0.39	3	0.37	7	0.55	−4	0.4
Used soap in handwashing demonstration	1	0.96	13	0.67	2	0.27	−13	0.24	−6	7.8
Report school latrines are usually very dirty	−16	0.44	−7	0.55	−49	**0.05**	−47	< **0.01**	−4	−0.5

* HP&WT schools received intervention with hygiene promotion and water treatment. Sanitation + HP&WT schools received the same, plus additional latrines.

† Percentage point change in school-aggregated values, adjusted for cluster sampling and unequal probability of pupil selection.

‡ *P* value of *t* test comparing difference from baseline to follow-up between intervention and control groups.
